# Supplementary material for: PPARγ Acetylation in Adipocytes Exacerbates BAT Whitening and Worsens Age-Associated Metabolic Dysfunction
Source: Cells. 2023 May 18;12(10):1424. doi: 10.3390/cells12101424 (PMC10217233; doi:10.3390/cells12101424)
Supplement: Supplementary file 1 [file cells-12-01424-s001.zip › cells-2362332-supplementary.pdf]

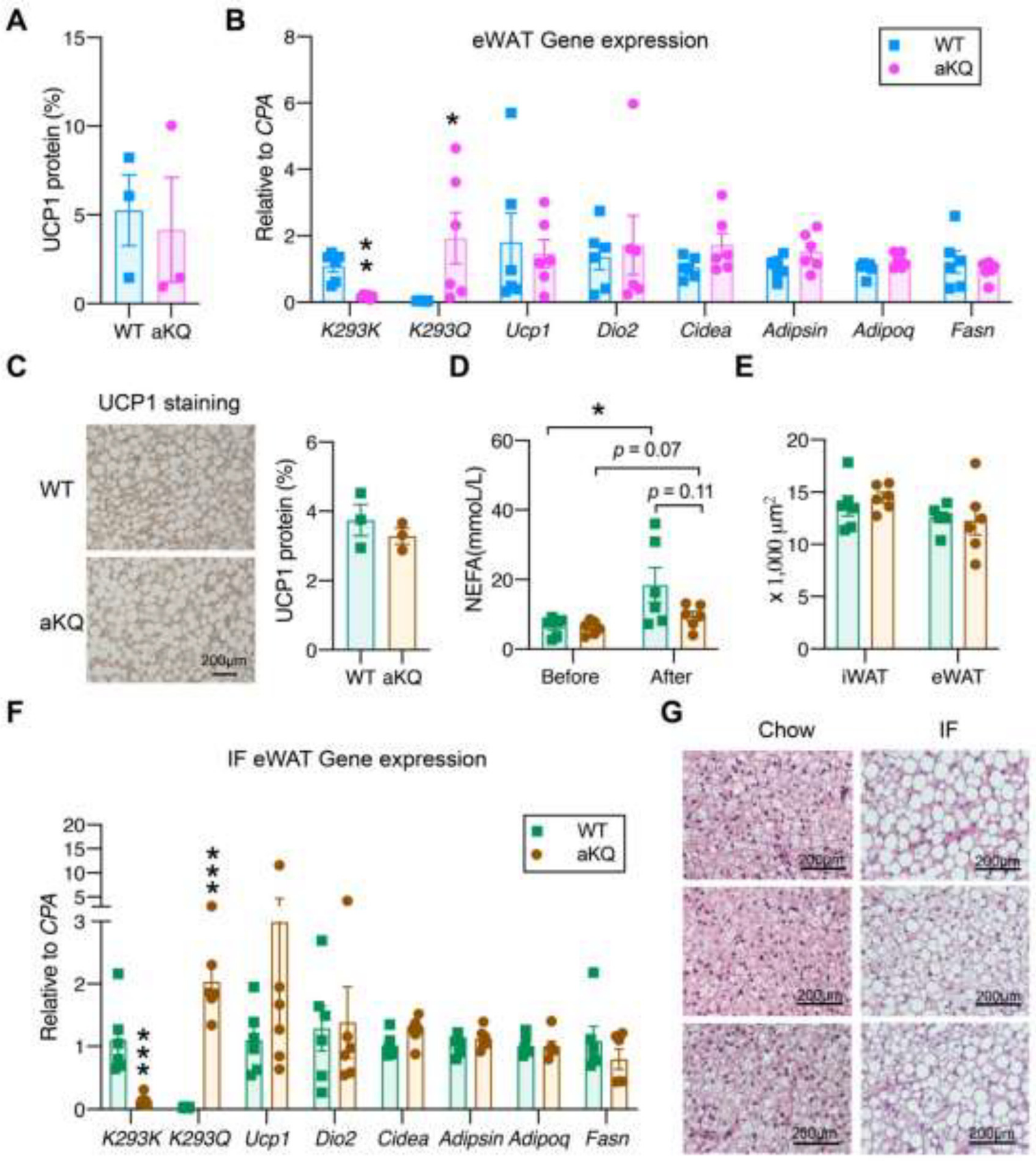

Figure S1. Metabolic characterizations in middle-aged mice. (A-B). Middle-aged male mice on chow diet feeding were sacrificed after an overnight fast. (A). Quantification of immunohistochemical staining of UCP1 protein in BAT of middle-aged mice on chow diet (n = 3, 3). (B). qPCR analysis of gene expression levels in eWAT (n = 6, 6). (C-G) Male middle-aged mice were subjected to intermittent fasting (IF) for 6 weeks. (C). Immunohistochemical staining and quantification of UCP1 protein in the BATs of WT and aKQ mice after IF (n = 3, 3). (D). Plasma NEFA levels before and 30 minutes after isoproterenol injection (10 mg/kg) (n = 6, 6). (E) Adipocyte sizes of WATs on IF (n = 6, 6). (F). qPCR analysis of gene expression in the eWATs of middle-aged mice on IF (n = 6, 6). (G). H&E staining of BAT in middle-aged mice before and after IF. * $p < 0.05$, ** $p < 0.01$, *** $p < 0.001$.
